# Increased expression of Matrix Metalloproteinase 9 in liver from NZB/W F1 mice received antibody against human parvovirus B19 VP1 unique region protein

**DOI:** 10.1186/1423-0127-16-14

**Published:** 2009-01-26

**Authors:** Chun-Chou Tsai, Bor-Show Tzang, Szu-Yi Chiang, Gwo-Jong Hsu, Tsai-Ching Hsu

**Affiliations:** 1 Institute of Immunology, Chung Shan Medical University, Taichung, Taiwan; 2 Institute of Biochemistry and Biotechnology, Chung Shan Medical University, Taichung, Taiwan; 3 Department of Health, Executive Yuan, Hua-Lien Hospital, Hua-Lien, Taiwan; 4 Division of Infectious disease and Department of Internal Medicine, Chia-Yi Christian Hospital, Chia-Yi, Taiwan

## Abstract

**Background:**

Human parvovirus B19 infection has been postulated to the anti-phospholipid syndrome (APS) in autoimmunity. However, the influence of anti-B19-VP1u antibody in autoimmune diseases is still obscure.

**Methods:**

To elucidate the effect of anti-B19-VP1u antibodies in systemic lupus erythematosus (SLE), passive transfer of rabbit anti-B19-VP1u IgG was injected intravenously into NZB/W F1 mice.

**Results:**

Significant reduction of platelet count and prolonged thrombocytopenia time were detected in anti-B19-VP1u IgG group as compared to other groups, whereas significant increases of anti-B19-VP1u, anti-phospholipid (APhL), and anti-double strand DNA (dsDNA) antibody binding activity were detected in anti-B19-VP1u group. Additionally, significant increases of matrix metalloproteinase-9 (MMP9) activity and protein expression were detected in B19-VP1u IgG group. Notably, phosphatidylinositol 3-phosphate kinase (PI3K) and phosphorylated extracellular signal-regulated kinase (ERK) proteins were involved in the induction of MMP9.

**Conclusion:**

These experimental results firstly demonstrated the aggravated effects of anti-B19-VP1u antibody in disease activity of SLE.

## Background

Human parvovirus B19 (B19) is known as a parvovirus of human pathogen [1] that consists two structural proteins including VP1 and VP2, which are identical except for the 227 amino acids at the amino-terminal end of the VP1-protein, the so-called VP1-unique region (VP1u) [2]. Recently, B19-VP1u has been reported to have the phospholipase A2 (PLA2) motif and secreted phospholipases A2 (sPLA2) activity [3-6], and is associated with various autoimmune diseases [7].

The infection of B19 has been postulated to the generation of various autoantibodies including anti-nuclear antibody (ANA), anticardiolipin antibody (aCL), and anti-phospholipid antibody (APhL) [7-11], as well as the anti-phospholipid syndrome (APS) [8]. Notably, a significant similarity existed in the specificity of APhL between patients with B19 infection or systemic lupus erythematosus (SLE) was reported [9,10]. Recent studies have suggested that B19 may exacerbate or even induce SLE [7,8]. Our recent findings indicated that serum from patients with acute B19 infection have a high frequency in recognition of cardiolipin (CL) and β2GPI, and the phospholipase domain observed in the B19-VP1u may contribute to the generation of APhL [12]. Additionally, the BALB/c mice immunized with anti-B19-VP1u IgG developed thrombocytopenia, prolongation of aPTT, and autoantibody against β2GPI and PhL and suggested the association among anti-B19-VP1u IgG and production of anti-β2GPI antibodies, APhL, and APS-like autoimmunity [13].

However, no further study was performed in elucidating the effect of anti-B19-VP1u antibody on disease activity in SLE. In current study, we treated NZB/W F1 mice with passive transfer of rabbit anti-B19-VP1u antibody to investigate the effect of elicited anti-B19-VP1u antibody on diseases activity in SLE.

## Methods

### Preparation of recombinant human B19-VP1 unique region protein and rabbit anti-B19-VP1 unique region antibody

Construction of B19-VP1u cDNA into pET-32a expression vector (Novagene, Cambridge, MA) was performed and the recombinant B19-VP1u protein was purified as described in our recent publication [12,13]. For generation of antisera against the B19-VP1u, four female New Zealand White rabbits were immunized subcutaneous in the neck region with 0.5 mg of purified recombinant B19-VP1u protein in Freund's complete adjuvant (Sigma, Saint Louis Mo, USA) followed by injection at two-week intervals with 0.25 mg of B19-VP1uproteininFreund'sincompleteadjuvant (Sigma, Saint Louis Mo, USA). A control group were immunized with Freund's complete adjuvant and followed by injection with Freund's incomplete adjuvant. All sera reacted specially with the B19-VP1u by immunoblotting analysis.

### Animals and induction of experimental APS by passive transfer

Twenty-four female NZB/W F1 mice at age of 8 weeks were purchased from National Taiwan University, Laboratory Animal Center, Taiwan and housed under supervision of the Institutional Animal Care and Use Committee at Chung Shan Medical University, Taichung, Taiwan. Induction of experimental APS by passive transfer was performed according to the method of Blank [14]. Disease activity of mice was determined by monitoring the proteinuria biweekly with Albustix test strips from the age of 14 weeks for ten weeks as described previously [15]. All rabbit IgG were isolated using Protein A beads as described in our recent report [13,16]. The dosage of anti-B19-VP1u IgG is based on a previous study of inducing APS in mice with anti-cardiolopin antibodies [14]. Proportionally, the common used concentration of immunoglobulins-preparations in mice is 20 ug/dosage and the titer of 20 ug rabbit anti-B19-VP1u IgG is similar to 27 U after determination and mathematics conversion (IBL-America, MN, USA) while the values of other control IgG preparations are less than 7 U. The titer greater than 12 U is considered as positive. The mice at age of 20 weeks were divided into four groups and were intravenously received rabbit anti-B19-VP1u IgG (20 ug), normal rabbit IgG (20 ug), rabbit anti-B19-NS1 IgG (20 ug), and PBS through the tail vein, respectively. The mice were then sacrificed on day 30 by CO_2 _asphyxiation and the heart blood samples were collected. APS clinical parameters, including thrombocytomenia and prolonged activated partial thromboplastin time [aPTT], were evaluated and the platelet counts were detected using Systemex (KX-21, KOBE, Japan). The presence of lupus anticoagulants were evaluated by the prolongation of aPTT in a mixing tests by adding 1 volume of plasma from whole blood mixed with sodium citrate 0.123 mol/l in a 9:1 ratio to 1 volume of each cephalin and incubating for 2 minutes at 37°C. Another volume of 0.025 M CaCl2 (Sigma, Saint Louis Mo, USA) was added, and the clotting time was recorded in seconds using Coatron M1 (TECO GmbH, Neufahrn NB, Germany).

### ELISA

Direct antigen-specific ELISA kits were used to detect APhL IgG (Louisville APL Diagnostics, Inc. GA, USA) and anti-dsDNA IgG (INOVA Diagnostics, Inc. CA, USA) was performed as described in our recent publication [12,13]. The color reaction was performed as described above. For detecting the binding activity of anti-B19-VP1u antibody, recombinant B19-VP1u was coated in a 96 well plate and ELISA was performed as described in our recent report [13]. The cutoff value for each ELISA experiment was obtained (mean+3SD) and the absorbance above the value is regarded as positive.

### Preparation of tissue extract and determination of protein

All procedures were performed at 4°C. Liver samples obtained from NZB/W F1 mice were homogenized in 600 ul PRO-PREP™ solution (iNtRON Biotech, Korea) by 30 strokes using a Dounce Homogenizer (Knotes Glass, Vineland, NJ). The homogenates were centrifuged at 13,000 rpm for 10 minutes at 4°C and the supernatant was stored at -80°C until use. Protein concentration of tissue extracts was determined according to the method described by Bradford [17] using bovine serum albumin as standards.

### Gel zymography

MMP-2 and MMP-9 activities were analyzed by gelatin-zymography assays as previously described [18]. Ten microliters of ten-fold diluted serum or 25 μg protein lysates of liver tissue from NZB/W F1 mice with passive transfer anti-idiotype antibodies were separated by an 8% Sodium dodecyl sulfate-polyacrylamide gel electrophoresis (SDS-PAGE) gels containing 0.1% gelatin. Gels were washed for 30 min in 2.5% Triton X-100 to remove the SDS and then soaked in the reaction buffer containing 40 mM Tris-HCl, pH8.0, 10 mM CaCl_2 _and 0.02% NaN3 for 30 min. The reaction buffer was changed to a fresh one, and the gels were incubated at 37°C for 24 h. Gelatinolytic activity was visualized by staining the gels with 0.5% Coomassie brillant blue R-250, destained with methanol-acetic acid water, and relative MMP levels were quantitated by a gel documentation and analysis system (Appraise, Beckman-Coulter, Brea, California, USA).

### Western blot

The loading sample for each lane of Western blot was a pool of four random selected mice of the same group. Protein samples were separated in 12.5 or 10% of SDS-PAGE and electrophoretically transferred to nitrocellulose membrane (Amersham Biosciences, Piscataway, NJ, USA) according to the method of Towbin [19]. After blocking with 5% non-fat dry milk in (PBS), antibodies against MMP2, MMP9, phosphatidylinositol 3-phosphate kinase (PI3K) and phosphorylated extracellular signal-regulated kinase 1/2 (p-ERK1/2), and actin (Upstates, Charlottesville, Virginia, USA) were diluted in PBS with 2.5% BSA and incubated for 1.5 hr with gentle agitation at room temperature. The membranes were then incubated with horseradish peroxidase (HRP) conjugated secondary antibody. Pierce's Supersignal West Dura HRP Detection Kit (Pierce Biotechnology Inc., Rockford, IL) was used to detect the antigen-antibody complexes. The blots were scanned and quantified by densitometry (Appraise, Beckman-Coulter, Brea, California, USA).

### Statistical analyses

The paired t test and one-way ANOVA were used to analyze for statistical significance. A P value < 0.05 was considered significant.

## Results

### Enhanced APS-like syndrome in NZB/W F1 by passive transfer of purified rabbit anti-B19-VP1u antibody

**Figure 1:**
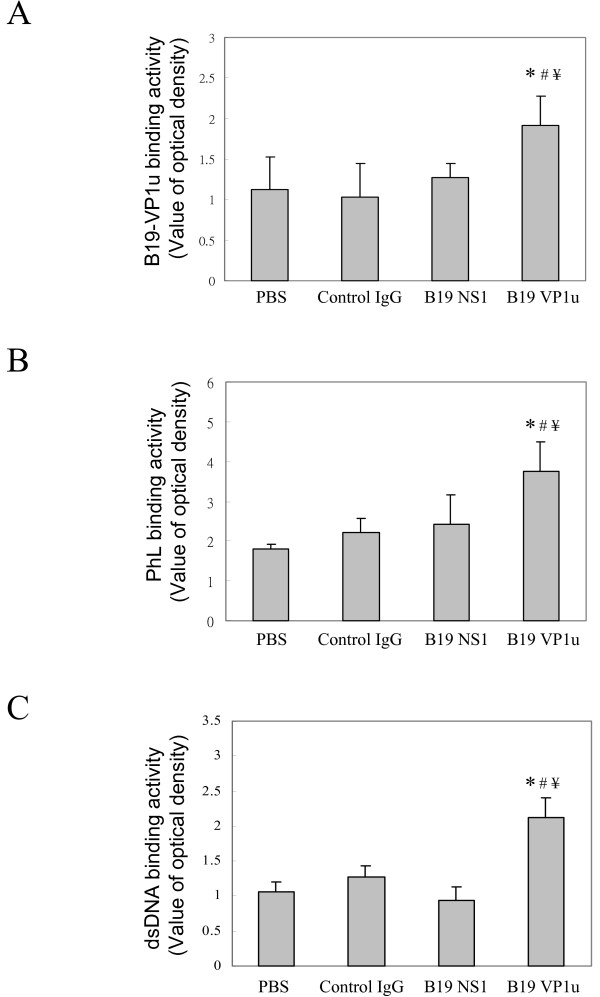
**Binding activity of various autoantibodies**. Anti-sera from the 4 groups of mice were analyzed for the binding activities of (A) B19-VP1u and (B) PhL, and (C) dsDNA. Bars represent the values of optical density. *, # and ¥ indicate significant differences as compared to the PBS, IgG, or B19-NS1 group, respectively. Similar results were obtained in three independent experiments.

To clarify the influence of anti-B19-VP1u antibody in disease activity and development of SLE, we employed and modified the experimental model as described in our previously report by immunizing NZB/W F1 mice intravenously with various kinds of purified rabbit IgG. The body weight and various clinical parameters including, WBC, RBC, HGB, HCT, MCV, MCH and MCHC revealed no significant variation in all groups of mice (Table 1). Notably, significant decreases of platelet counts and aPTT were observed in sera from NZB/W F1 mice that were received purified rabbit anti-B19-VP1u IgG, compared to those mice that were received normal rabbit IgG, rabbit anti-B19-NS1 IgG, or PBS, respectively (Table 1). Additionally, ELISA experiments were performed to elucidate the effect of anti-B19-VP1u IgG by analyzing the binding activities of APhL antibodies in NZB/W F1 mice that were received purified rabbit anti-B19-VP1u IgG. Elevated titers of anti-B19-VP1u and APhL and anti-dsDNA antibodies were detected in serum from NZB/W F1 mice that were received purified rabbit anti-B19-VP1u IgG as compared to those mice that were received normal rabbit IgG, anti-B19-NS1 IgG, or PBS, respectively (Fig. 1).

**Table 1:** Mice infused with various rabbit antibody or reagent

	Antibodies or reagent infused into mice			
	
	PBS [n = 6]	Control IgG [n = 6]	B19-NS1 [n = 6]	B19-VP1u [n = 6]
Body Weight (gram)	33.9 ± 1.7	33.9 ± 0.2	32.6 ± 2.5	33.6 ± 2.7
WBC (10^3 ^cells/ul)	6.3 ± 0.7	5.5 ± 1.1	6.2 ± 1.6	4.3 ± 1.4
RBC (10^6 ^cells/ul)	7.7 ± 0.6	8.7 ± 0.2	8.1 ± 0.2	7.8 ± 0.2
HGB (g/dL)	12.5 ± 1.1	15 ± 0.3	13.6 ± 0.5	13.3 ± 0.6
HCT (%)	44.2 ± 1.3	53.3 ± 2.2	50.4 ± 1.9	47.2 ± 1.4
MCV (fL)	57.3 ± 2.5	61.1 ± 1.1	62.3 ± 0.8	60.4 ± 0.9
MCH (pg)	16.2 ± 0.3	17.2 ± 0.2	16.9 ± 0.3	17.4 ± 0.6
MCHC (g/dL)	28.3 ± 1.7	28.2 ± 0.8	27.1 ± 0.2	28.3 ± 1.3
aPTT (second)	69.5 ± 8.1	76.4 ± 11.6	87 ± 6.0	156 ± 27.0* ^,#,¥^
Platelet count (10^3 ^cells/mm^3^)	1186 ± 219	1198 ± 124	1374 ± 179	808 ± 90.0* ^,#,¥^

The unit of the parameter is presented in bracket.

NS1: non-structure protein 1; VP1u: VP1 unique region protein; WBC: white blood cell; RBC: red blood cell; HGB: Hemoglobin; HCT: Hematocrit; MCV: Mean corpuscular volume; MCH: mean corpuscular hemoglobin; MCHC: hemoglobin concentration; aPTT: activated partial thromboplastin time.

*, #, and ¥ indicate P < 0.05, as compared to the PBS, control IgG, or B19-NS1 group, respectively.

### Enhanced MMP-9 activity and expression in liver from NZB/W F1 by passive transfer of purified rabbit anti-B19-VP1u antibody

**Figure 2:**
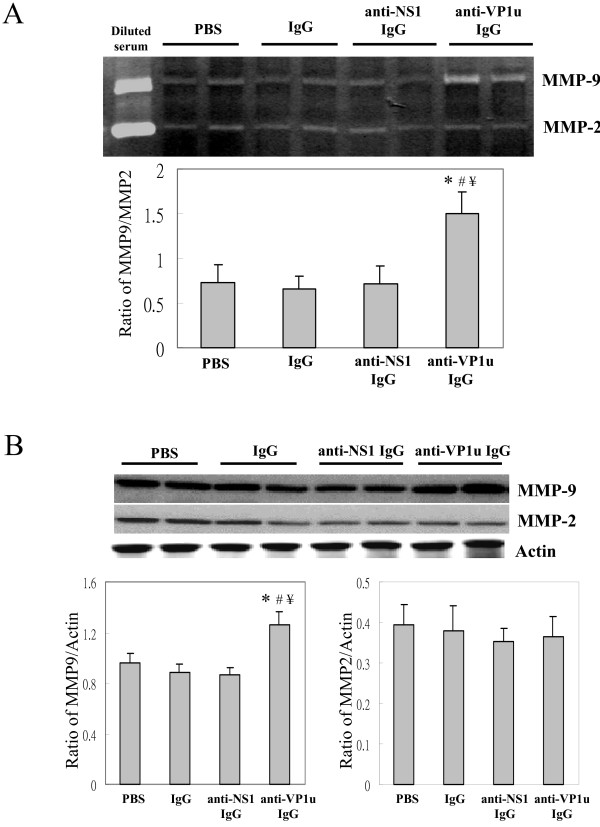
**Activities and protein expression of MMP-9 andMMP-2**. Liver samples obtained from the 4 groups of mice were analyzed for the (A) MMP-9 and MMP-2 activity and (B) MMP-9 and MMP-2 protein expression. The quantified results of MMP-9/MMP-2, MMP-9/actin, and MMP-2/actin ratio were shown in the lower panels, respectively. *, # and ¥ indicate significant differences as compared to the PBS, IgG, or B19-NS1 group, respectively. Similar results were obtained in three independent experiments.

To further examine the effect of anti-B19-VP1u antibody on pathogenesis of liver in NZB/W F1 mice, MMPs activity and protein expression were examined. Significant increase of MMP9 activity was observed in liver of NZB/W F1 mice that were received rabbit anti-B19 VP1u IgG as compared to PBS, Control IgG, or B19-NS1 IgG group, respectively (Fig. 2A). However, no significant variation was detected in MMP-2 activity among all experimental groups (Fig. 2A). The quantified results of MMP-9/MMP-2 ratio were shown in lower panel of Fig 2A. Moreover, Western blots were performed to examine the expression of MMP9 and MMP2. Significant increase of MMP-9/MMP-2 ratio was detected in B19-VP1u group as compared to PBS, Control IgG, or B19-NS1 IgG group, respectively (Fig 2B). Quantified results were shown in the lower panel of Fig 2B.

### Increased phosphorylation of ERK 1/2 and PI3K proteins in NZB/W F1 by passive transfer of purified rabbitanti-B19-VP1u antibody

**Figure 3:**
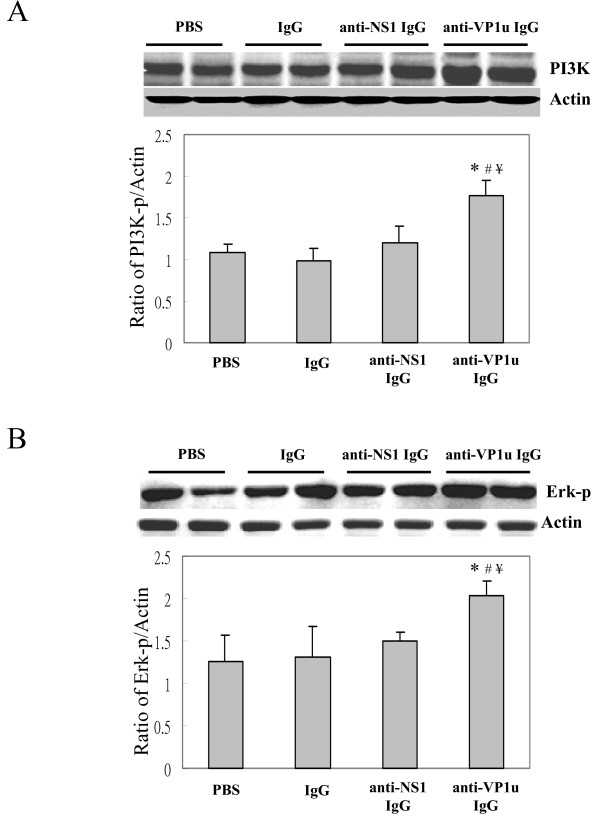
**Presence of PI3K and phosphorylated ERK proteins**. Liver samples obtained from the 4 groups of mice were analyzed for the (A) PI3K and (B) phosphorylated ERK (ERK-p) proteins. The quantified results of PI3K/actin and ERK-p/actin ratios were shown in the lower panels, respectively. *, # and ¥ indicate significant differences as compared to the PBS, IgG, or B19-NS1 group, respectively. Similar results were obtained in three independent experiments.

To clarify the possible signaling pathway involved in the activation of MMP9 by B19-VP1u, various signaling molecules including, PI3K, Erk1/2-p, p38-p, and JNK-p were examined. Notably, the PI3K and phosphorylation of ERK 1/2 proteins were observed in liver from NZB/W F1 mice that were treated with rabbit anti-B19 VP1u IgG, as compared to PBS, Control IgG, or B19-NS1 IgG group, respectively (Fig 3). However, no significant variations of p38-p and JNK-p were observed in all experimental groups (data not shown). Quantified results were shown in lower panels of Fig. 3A and 3B.

## Discussion

B19 infection has been implicated as a trigger of various autoimmune diseases including the induction of autoantibodies in patients with SLE [7-11]. However, the roles of B19-VP1u and anti-B19-VP1u antibody in pathogenesis of autoimmune diseases remain unclear. In current study, we demonstrated the aggravated effect of anti-B19-VP1u antibody on disease activity in SLE by analyzing the APS-like syndrome. Significant aggravated disease activities, including reduced platelet count, prolonged thrombocytopenia time, increased binding activity of autoantibody, elevated MMP9 activity and protein expression, were observed in NZB/W F1 mice that were received anti-B19-VP1u IgG as compared to those mice that were received rabbit control IgG, rabbit anti-B19-NS1 IgG or PBS, respectively.

APS-like syndrome is recognized as a striking analogy between the clinical features and hematologic findings in patients with SLE or B19 infection [7-11]. APS is characterized by raised titers of circulating APhL that bind target molecules primarily via β2GPI, and/or lupus anticoagulant in association with recurrent fetal loss, thromboembolic phenomena, thrombocytopenia, CNS, heart, and other organ involvement [20,21]. β2GPI shares a common amino acid sequence with various microbial pathogens, and may be the cause of APS or production of the cross reacting autoantibodies [22-24]. Our previous study identified four cases of B19 infection associated with the production of aCL and anti-β2GPI antibodies [12]. Consistently, our recent study has demonstrated a spectrum of experimental APS-like autoimmunity induced by passive transfer of purified rabbit anti-B19-VP1u IgG antibodies and provided a connection between anti-B19-VP1u IgG and pathogenesis of SLE [13]. In current study, we indicated the significant increases of APS-like syndromes including increased platelet count, prolonged PTT, and increased binding activity of APhL and dsDNA in NZB/W F1 mice that were received purified anti-B19-VP1u IgG. These findings strikingly suggested the aggravated effect of anti-B19-VP1u IgG in SLE.

Numerous studies have suggested that B19-VP1u plays a crucial role in induction of anti-β2GPI and APhL antibodies [8,9,13]. Adsorption experiment revealed the partially reduced reactivity of anti-B19-VP1u antibody to CL and β2GPI [12], suggesting similar epitopes or conformation may exist between B19-VP1u, CL and β2GPI [8,9,12,13]. These experimental results may also account for the cross-reactivity of anti-B19-VP1u antibody against CL, β2GPI, and ph ospholipid and suggest underlying mechanisms in development of APS-like syndrome such as APhL antibody. In current study, significant increase of APhL antibodies was observed in NZB/W F1 mice that were received purified rabbit anti-B19-VP1u IgG, as compared to those mice that were received control rabbit IgG, rabbit anti-B19-NS1 IgG or PBS, respectively. This finding may be due to the similar epitopes and anti-idiotype networks among anti-B19-VP1u IgG, and APhL antibody. However, the underlying mechanism is still unclear and deed merited further investigations.

Previous studies have postulated MMPs to the pathogenesis of SLE [25-28]. Cleavage of myelin basic protein or type II gelatins by MMP-9 will produce remnant epitopes and contribute to the development of autoimmunity [27,28]. In recent studies, elevated MMP-9 activity was founded in both human and mice model with SLE [28-31] and recognized to play crucial roles in development of SLE. Additionally, various studies have indicated the involvement of ERK and PI3K in activation of MMP-9. In monocytes from patients with rheumatoid arthritis, inhibition of extracellular signal-regulated kinase (ERK) abolished Cyclosporine A-induced MMP-9 expression [32]. Another study reported that PI3K/Akt activation promotes transcriptional co-factor p300 recruitment and activation and led to increased proMMP-9 expression in rat astrocyte [33]. In current study, significant increases of MMP-9 activity and protein expression were detected in NZB/W F1 mice that were received anti-B19-VP1u IgG, as well as the increased PI3K and phosphorylated ERK proteins. These data suggest the aggravated effect of anti-B19-VP1u IgG in pathogenesis of SLE and the involvement of activation of MMP-9 via PI3K and ERK signaling pathway. It could provide clues in treatment of SLE by inhibiting PI3K and EKR signaling pathway.

## Conclusion

Taken together, our experimental results firstly demonstrated the aggravated APS-like syndromes in NZB/W F1 mice that were received anti-B19-VP1u IgG. Additionally, it could provide clues in understanding the roles of anti-B19-VP1u IgG in SLE and suggest possible therapeutic potential by inhibiting PI3K or AKT signaling pathway.

## Competing interests

The authors declare that they have no competing interests.

## Authors' contributions

CCT performed the animal study, ELISA, zymography, and Western blotting. BST conceived this study, drafted the manuscript, and performed the performed statistical analyses. SYC and GJH provided material support and encouragement for this work. TCH provided material support and direction, and drafted significant portions of the manuscript.
